# Najm transconal unroofing and left anterior descending exteriorization with intraventricular course

**DOI:** 10.1016/j.xjtc.2025.11.004

**Published:** 2025-11-19

**Authors:** Kaoutar Farahi, Praise Chovwen, Munir Ahmed, John P. Costello, Nicholas Szugye, Shinya Unai, Hani K. Najm

**Affiliations:** a Department of Pediatric and Congenital Heart Surgery, Cleveland Clinic, Cleveland, Ohio; b Department of Adult Cardiac Surgery, Cleveland Clinic, Cleveland, Ohio; c Case Western Reserve University School of Medicine, Cleveland, Ohio

**Figure:**
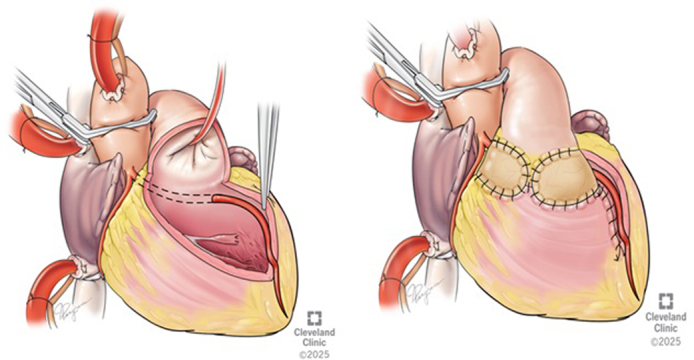
Combined Najm transconal unroofing with LAD exteriorization.

**Central Message**

LAD arising from the RCA with a deeply seated septal and intraventricular course can be safely and reproducibly exteriorized using transconal unroofing with full-length RV course exteriorization.

Anomalous origin of the left anterior descending artery (LAD) from the right coronary artery (RCA) with a transseptal course and multiple myocardial bridging to the apex represents a rare but complex coronary anomaly. It carries a risk of ischemia and poses technical challenges as the result of its complex myocardial trajectory. We describe a novel approach combining our previously described transconal unroofing and full-length LAD exteriorization, offering a safe and effective solution in this setting.

## Case Report

**Figure 1:**
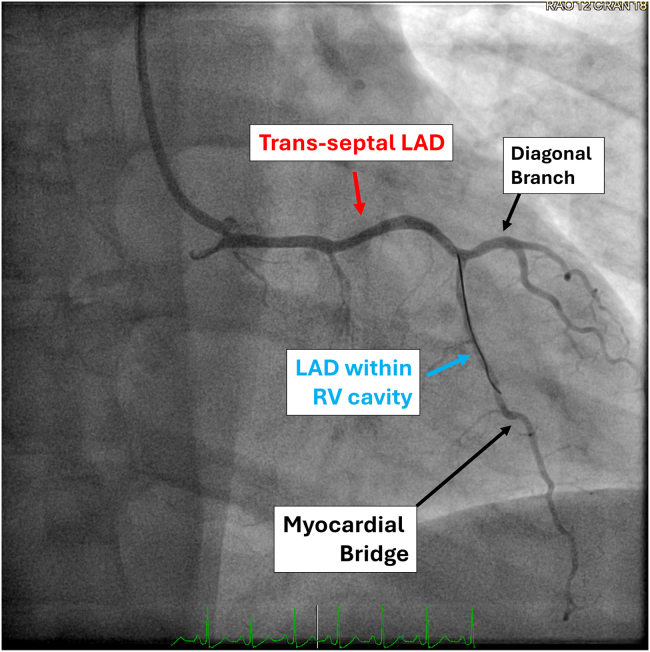
Coronary angiography demonstrating an anomalous left anterior descending artery (*LAD*) with a transseptal and intracavitary course. The LAD originates from the right coronary artery (*RCA*) and traverses the interventricular septum, entering the right ventricular (*RV*) cavity before re-emerging on the anterior surface. A distinct myocardial bridge segment is visualized distally. The diagonal branch arises normally from the LAD. Labels highlight the transseptal LAD segment (*red*), LAD within the RV cavity (*blue*), and myocardial bridge (*black*).

A 42-year-old woman with a medical history of hypertension and tobacco use presented with exertional chest pain. Noninvasive testing with single-photon emission computed tomography myocardial perfusion imaging was inconclusive. Coronary computed tomography angiography revealed an anomalous LAD originating from the RCA with a transseptal and right intraventricular course. The LAD coursed through the interventricular septum and did not emerge as usual to the surface of the epicardium, rather stayed within and traversed the right ventricular (RV) cavity, and was roofed with multiple deep myocardial bridging until it emerged at apex. Invasive coronary angiography confirmed the anomalous origin. Functional assessment using resting full-cycle ratio demonstrated a value of 0.60 distal to the bridged segment, consistent with hemodynamically significant dynamic obstruction (Figure 1). Institutional review board approval was not required. Informed consent was obtained from the patient, including patient consent for the publication of study data and accompanying images.

### Operative Interventions

A median sternotomy was performed. Cardiopulmonary bypass was initiated with distal aortic and bicaval cannulation (bypass time 89 minutes, crossclamp time 81 minutes). After aortic crossclamping, myocardial arrest was achieved using blood cardioplegia delivered through the aortic root, and additional low-flow root cardioplegia was administered during unroofing to fill the LAD and facilitate safe dissection. The technique of transconal unroofing was described in our previous publication.^1^ Dissection was carried out in the plane between the aorta and right ventricular outflow to visualize the LAD. The right ventricular outflow tract (RVOT) was opened transversely, exposing the intramyocardial LAD, which was deeply embedded in muscle throughout its septal course.

**Figure 2:**
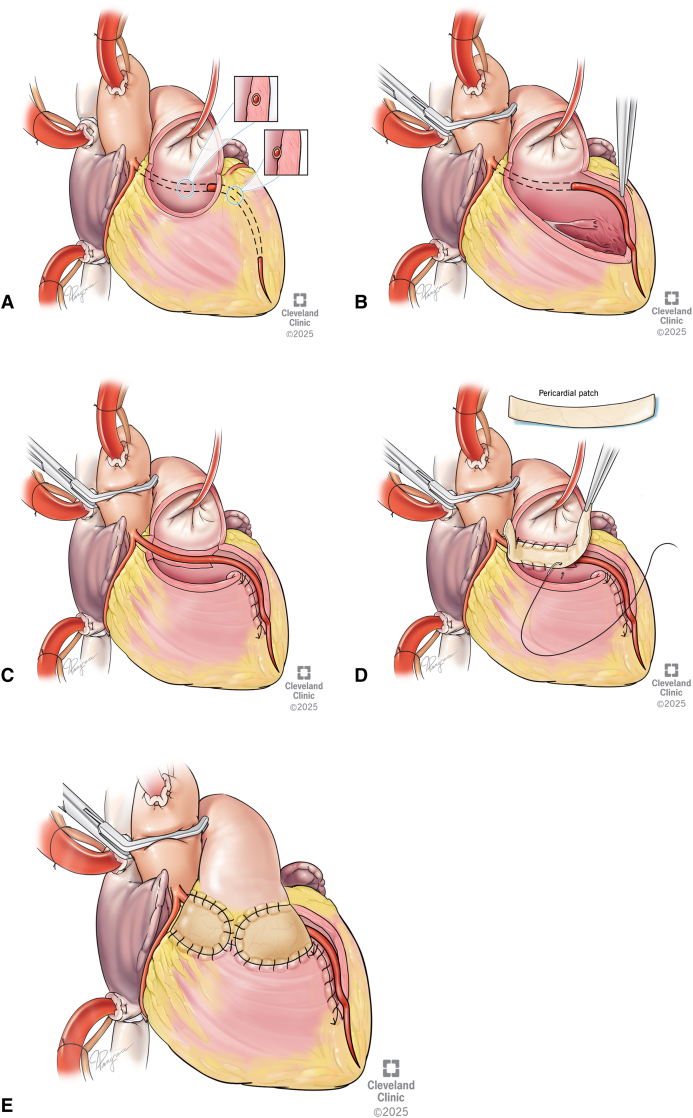
A, Artist depiction of the anteriorly opened right ventricular outflow tract (*RVOT*) and the appearance of the left anterior descending artery (*LAD*) underneath the septal muscle and running within the cavity of the right ventricle to the apex. The *dotted lines* indicate the planned incision line to unroof the LAD. Inset (*right*) demonstrates the sagittal view of the anomalous LAD, surrounded by muscle. B, Artist depiction viewed through the anterior opening in the RVOT. The coronary segment is unroofed throughout the entire intraseptal area. The unroofing was extended distally to the apex as it emerged. C, After transeptal unroofing, the free wall of the right ventricle was folded in and sutured to the septum few millimeters away from LAD. D, RVOT reconstruction was performed using a patch of autologous pericardium. E, Artist depiction demonstrating the completed repair, with the anterior right ventricular outflow tract (*RVOT*) closed with pericardium and the LAD entirely exteriorized.

A transconal unroofing technique was used to unroof the LAD free from its muscular bed. As we followed the LAD, it was clear that the course was within the cavity of the right ventricle and in between the trabeculae. The unroofing was extended distally along 7 cm to the apex as it emerged. Once unroofed, the free wall of the right ventricle, now free from septal attachment, was folded in and sutured to the septum few millimeters away from the LAD, leaving the LAD entirely exteriorized. RVOT reconstruction was performed using a patch of autologous pericardium, secured circumferentially with 5-0 PROLENE (Ethicon) sutures, and positioned to avoid tension or compression over the LAD, as described in our publication (Figure 2).

**Figure 3:**
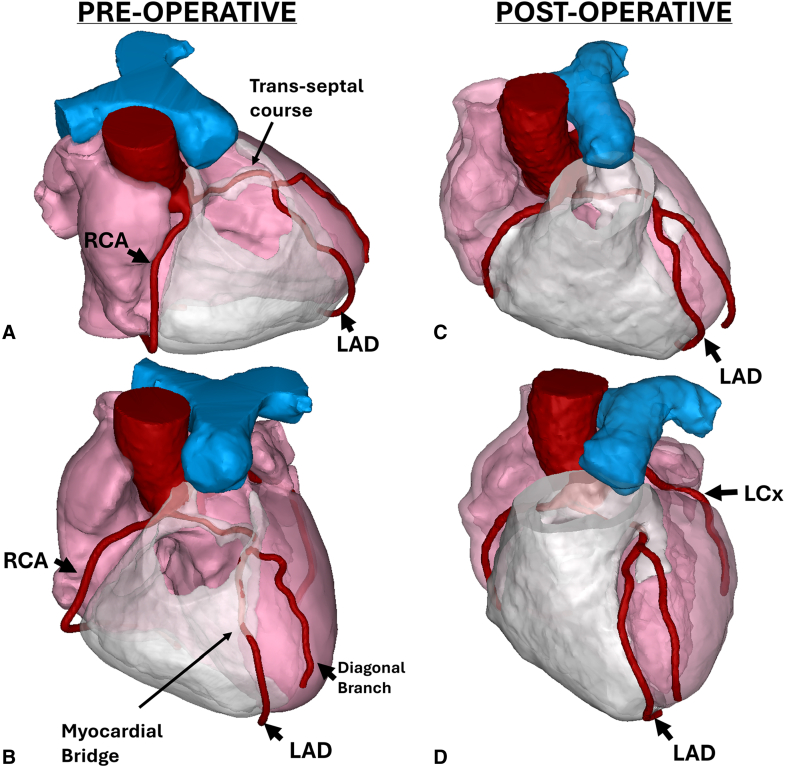
Three-dimensional computed tomographic (*CT*) reconstructions are shown illustrating preoperative and postoperative anatomy of an anomalous left anterior descending artery (*LAD*) with a transseptal and intracavitary course through the right ventricle (*RV*) and associated myocardial bridge. A and B (preoperative), The LAD originates from the right coronary artery (*RCA*) and follows a transseptal and intracavitary course within the RV cavity before emerging on the anterior surface. C and D (postoperative), After transconal unroofing and full length LAD exteriorization, the LAD is fully released, with restoration of a normal epicardial course. The left circumflex artery (*LCx*) arises normally from the left coronary sinus.

Hemostasis was assured, and the crossclamp was removed. The heart resumed sinus rhythm without inotropic support. Transesophageal echocardiography showed preserved biventricular function with no regional wall motion abnormalities. She was discharged on postoperative day 5. At follow-up, she remained chest pain free (Figure 3).

## Discussion

Anomalous origin of the LAD from the RCA is a rare coronary anomaly, occurring in less than 0.1% of general population.^2^ The condition becomes particularly complex when accompanied by a deep myocardial bridge in particular when it runs in the right ventricular cavity. This constellation of anatomical features markedly increases the risk of ischemia and significant technical challenges for surgical management.

Our patient presented with a transseptal and intraventricular course of the LAD, accompanied by extensive myocardial bridge from the proximal to distal segments. This anatomical configuration of anomalous aortic origin of the left coronary artery can be associated with dynamic compression and ischemia.^1^ Conventional surgical strategies, such as coronary artery bypass grafting (CABG), are often suboptimal in this setting because of competitive flow and a high incidence of graft failure.^3^

Our surgical approach involved transconal unroofing of a transseptal LAD course, relief of a 7-cm myocardial bridge extending to the apex, and repair of the right ventricular wall. This technique entails complete unroofing of the intramyocardial segment and reconstruction of the right ventricular outflow tract.^4^

Several alternative surgical strategies for managing unusual LAD courses have been reported. Early series of intracavitary coronary arteries encountered during CABG described management with buttressed mattress closure, arterial repositioning of the vessel.^5^ Ventriculotomy repair with pledgeted horizontal mattress sutures has also been described to approximate the RV wall to the septum when an intracavitary LAD is exposed.^E1^ More recently, a case associated with an anomalous RCA described a LAD with a short 3-cm myocardial bridge that communicated with the RV cavity through trabeculated sinusoids, which was managed by suturing the RV free wall to the septum with pledgeted sutures.^E2^

In contrast, our patient had an anomalous LAD originating from the RCA with transseptal and 7-cm intraventricular course extending to the apex, entirely roofed by deep myocardial bridge. This constellation required a combined strategy: transconal unroofing of the proximal segment, extended exteriorization of the LAD through the RV cavity, folding of the RV free wall inward and suturing it to the septum a few millimeters below the artery, and RVOT patch reconstruction. Using this approach, we achieved a safely reproducible full-length exteriorization while preserving RV geometry and ensuring unobstructed coronary flow.

In the largest multicenter analysis of anomalous aortic origin of a coronary artery conducted by the Congenital Heart Surgeons' Society, features such as intramural or transseptal courses and slit-like orifices were significantly associated with myocardial ischemia and sudden cardiac events.^E3^ Although transseptal variants are not traditionally categorized as “malignant,” published reports indicate that they may still be clinically significant in selected patients.^E4^^,^^E5^ Our patient demonstrated a combination of these adverse morphologic features, which together provided the rationale for surgical intervention.

Other surgical strategies have been described to address the transeptal course of the left main coronary artery, including unroofing of the overlying muscle fibers with LeCompte maneuver,^E6^ pulmonary root translocation,^E7^ and surgical reimplantation.^E8^

Our recent reports have demonstrated that transconal unroofing combined with RVOT patch reconstruction provides excellent early outcomes, with improvement in functional status and no mortality at midterm follow-up,^4^ Reconstruction of the RVOT is performed using a customized autologous pericardial patch. The shape and orientation of the patch are carefully tailored, particularly in cases with an early takeoff of the LAD or extensive myocardial bridging.^3^ In our approach, special attention was paid to preserving septal branches and maintaining native right ventricular geometry during closure. Postoperative transthoracic echocardiography confirmed preserved right ventricular systolic function, with no evidence of regional wall motion abnormalities.

Although anomalous coronary arteries have not been definitively associated with accelerated atherosclerosis, LAD anomalies tend to present earlier and more symptomatically, highlighting their functional burden even in the absence of fixed obstructive disease.^E9^ These findings further support the importance of recognizing and addressing anatomical variants such as myocardial bridges and deep intraventricular coronary courses.

## Conclusions

This report demonstrates that even an LAD deeply embedded within the interventricular septum and right ventricular cavity to the apex can be safely and completely exteriorized. This approach should be considered in symptomatic patients with anomalous LAD arising from the right coronary artery, particularly when associated with extensive myocardial bridge and a deep trans-septal course and in cases of an LAD diving in the RV cavity during CABG.

## Conflict of Interest Statement

The authors reported no conflicts of interest.

The *Journal* policy requires editors and reviewers to disclose conflicts of interest and to decline handling or reviewing manuscripts for which they may have a conflict of interest. The editors and reviewers of this article have no conflicts of interest.
